# Trends in International Cancer Research Investment 2006-2018

**DOI:** 10.1200/GO.20.00591

**Published:** 2021-04-28

**Authors:** Rachel Abudu, Gauthier Bouche, Karima Bourougaa, Lynne Davies, Kalina Duncan, Carla Estaquio, Anna Diaz Font, Marc S. Hurlbert, Paul Jackson, Linda Kroeskop-Bossenbroek, Ian Lewis, Giota Mitrou, Abdul Mutabbir, Christopher A. Pettigrew, Lynn Turner, Annemarie Weerman, Kari Wojtanik

**Affiliations:** ^1^ London School of Hygiene and Tropical Medicine, London, United Kingdom; ^2^ Anticancer Fund, Brabant, Belgium; ^3^ Institut National du Cancer, Boulogne-Billancourt, France; ^4^ International Cancer Research Partnership, Cardiff, United Kingdom; ^5^ US National Cancer Institute, Bethesda, MD; ^6^ World Cancer Research Fund International, London, United Kingdom; ^7^ Melanoma Research Alliance, Washington, DC; ^8^ Cancer Australia, Surry Hills, NSW, Australia; ^9^ Dutch Cancer Society, Amsterdam, the Netherlands; ^10^ National Cancer Research Institute, London, United Kingdom; ^11^ National Breast Cancer Foundation, Sydney, NSW, Australia; ^12^ Worldwide Cancer Research HQ, Edinburgh, United Kingdom; ^13^ Susan G. Komen, Dallas, TX

## Abstract

The International Cancer Research Partnership (ICRP) is an active network of cancer research funding organizations, sharing information about funded research projects in a common database. Data are publicly available to enable the cancer research community to find potential collaborators and avoid duplication. This study presents an aggregated analysis of projects funded by 120 partner organizations and institutes in 2006-2018, to highlight trends in cancer research funding. Overall, the partners’ funding for cancer research increased from $5.562 billion (bn) US dollars (USD) in 2006 to $8.511bn USD in 2018, an above-inflation increase in funding. Analysis by the main research focus of projects using Common Scientific Outline categories showed that Treatment was the largest investment category in 2018, followed by Early Detection, Diagnosis, and Prognosis; Cancer Biology; Etiology; Control, Survivorship, and Outcomes; and Prevention. Over the 13 years covered by this analysis, research funding into Treatment and Early Detection, Diagnosis, and Prognosis had increased in terms of absolute investment and as a proportion of the portfolio. Research funding in Cancer Biology and Etiology declined as a percentage of the portfolio, and funding for Prevention and Control, Survivorship and Outcomes remained static. In terms of cancer site–specific research, funding for breast cancer and colorectal cancer had increased in absolute terms but declined as a percentage of the portfolio. By contrast, investment for brain cancer, lung cancer, leukemia, melanoma, and pancreatic cancer increased both in absolute terms and as a percentage of the portfolio.

## BACKGROUND

Collaboration between research funding organizations is becoming increasingly important at an international level, to allow coordination of investment in common identified priority areas, reduce duplication, and fast-track outcomes. The International Cancer Research Partnership^1^ (ICRP) is an alliance that in 2021 includes more than 140 cancer research organizations from the United States, Canada, Europe, Japan, and Australia. ICRP maintains the only public source, worldwide, of current and past grants, totaling more than $80bn US dollars (USD) in cancer research funding since 2000. ICRP member organizations submit project-level data for their research portfolios to the ICRP database^2^ including PI name, host institution, city, country, funding organization, project title, abstract, start date, end date, and total funding amount for that period. Each project in the database is assigned to one or more cancer sites and research types. The research type classification (Common Scientific Outline or CSO) includes 34 codes, grouped into six categories (Biology, Etiology, Prevention, Early Diagnosis and Prognosis, Treatment, and Survivorship and Cancer Control).^3^ All fields (with the exception of project funding amount) are visible on the ICRP public website, and project funding amount is visible to partners who contribute data. The database includes current and historic projects, enabling researchers to identify potential collaborators and to avoid duplicating previous or existing research. The primary objective of this analysis was to assess if investment in types of research (CSO) and cancer sites had changed significantly between 2006 and 2018. As it was not possible to present detailed trends for all cancer sites within this paper, secondary objectives were to assess trends in investment in CSO categories for a cancer site with the highest percent increase in funding during this timeframe (pancreatic cancer) and to look at detailed trends in research types with the lowest investment levels, namely, prevention (CSO 3) and survivorship (CSO 6). This analysis and the accompanying data pack were produced to enable the partners, and the wider cancer research funding community, to monitor changing patterns in research investment internationally and to inform future strategic planning.

**CONTEXT**

**Key Objective**
This study provides, for the first time, an in-depth view of the evolving landscape of global cancer research funding from 2006 to 2018, based on project-level data provided by cancer research funding organizations classified by cancer type and research type.
**Knowledge Generated**
The results show that funding for cancer research shifted to be more translational and clinical in focus. Funding for all major cancer types increased between 2006 and 2018, and this study highlights that additional funding was allocated to cancers of high incidence or mortality.
**Relevance**
This study highlights gap areas for future research to enable strategic, focused research and funding decisions.

## METHODOLOGY

The ICRP database includes projects across the spectrum of cancer research. Projects within the database are for hypothesis-driven research or resources and infrastructure to support these. Some cancer research investments by funding partners (eg, capital expenditure on buildings for cancer research) are not included. Calendar Years (CY)2006-CY2018 were selected for this analysis, as project-level data were complete for all of the largest ICRP member funding organizations or consortia during this timeframe. In particular, to avoid sampling bias, CY2006 was selected as the start year because changes to project and/or award reporting at US National Institutes of Health (NIH) in FY07 are reflected in the ICRP database only from CY2006 onward. Figures for NIH investment in CY2005 and earlier in the ICRP database were not comparable with those for CY2006 and later and could have given rise to artificially low figures for CY2005, thus over-representing the increase for 2018. For the organizations included in this analysis, in 2018, the majority of $8,396 million (M) USD investment came from organizations that had also been in existence in 2006–data for these organizations were included in full for the period 2006-2018. Nearly $115M USD of the 2018 investment came from organizations whose cancer research programs had started after 2006, thereby representing real new investment in cancer research during this timeframe. Details of projects active in CY2006-CY2018 (inclusive) were extracted from the database and project dollars per CY calculated based on budget start and end dates, and days in a specific calendar year. Cancer site and CSO investments were calculated using percent allocations per project to avoid double accounting. All project funding amounts that were not in USD were converted to USD using the average CY2018 exchange rate, to avoid confounding underlying trends with those based on year-on-year currency fluctuations. Trends in CSO or cancer site investment were calculated using proportional annual investment, and significance was assessed using regression lines and *R*^2^ values. Statistical analysis suggested that polynomial regressions provided the best fit for the data because most trends were not linear.

Normalization of investment figures means that the figures presented here differ from those in individual partner organizations' annual reports, which may represent fiscal years or other accounting conventions. A top-level assessment of the effect of inflation over 2006-2018 was calculated using the CPI Inflation calculator.^4^

All projects in the portfolio were coded to one or more cancer sites and CSO categories. Cancer sites used by ICRP^3^ consist of 61 site codes, linked to one or more neoplasm codes in the International Classification of Diseases-10.^5^ Percent investment in site-specific cancers was compared with global cancer incidence and mortality rates for the same sites for 2018, sourced from the Global Cancer Observatory.^6^

In 2014, the ICRP partners migrated to a new version of the CSO (v2) and all earlier projects in the database were re-coded. As part of this migration, a statistical assessment of inter-rater reliability was conducted for both the CSO and cancer sites. Agreement was in the very good range for major CSO categories (Cohen's kappa coefficient 0.85) and cancer sites (Cohen's kappa: 0.80) and in the good range for minor categories of the CSO (Cohen's kappa: 0.69).^7-9^

In addition, projects that were wholly or partially relevant to childhood, adolescent, and young adult (CYA) cancers were tagged. Project dollars from organizations that exclusively funded CYA cancers were included in full. Investment in CYA projects from organizations funding research in both childhood and adult cancers was calculated using a combination of the CYA cancer project tag and a filter to exclude any cancer site investment that was not common in childhood, to avoid overstating investment in CYA cancers.

## RESULTS

### Trends in the Overall Portfolio

Over the 10-year period CY2006-CY2018, research investment by ICRP partner organizations increased from $5.5bn USD to $8.5bn USD and funded project numbers increased from 34,305 in 2006 to 44,131 in 2018. This increase in investment to 2018 was a real increase in funding of 24% above inflation. Most of the increase resulted from additional investments of more than $2.9bn USD by partner organizations that were in existence in 2006, with a substantial additional contribution (nearly $115M USD) from new organizations founded since 2006 and whose investment in cancer research programs started after 2006. Internationally, the government sector was the biggest funder of cancer research (90%) in 2006 and its contribution to overall funding was similar in 2018 (93%). During CY2009-CY2012, there was a peak in funding (not shown), largely attributable to additional investments of the US Government's American Recovery and Reinvestment Act (ARRA), which added around $0.5-$1bn USD per annum to research investment during this period, primarily to projects in the United States.

The analysis included data from 120 funding organizations with active research projects during the time period selected for the analysis. The largest contributor ($7.3bn USD in 2018) to the overall portfolio was the US NIH, including all investment from the National Cancer Institute and relevant research from other NIH Institutes, as defined by the NIH’s internal cancer spend category. Other organizations contributed research totaling $1.2bn USD in 2018 to the analysis.

### Trends by Type of Research (CSO) and Cancer Site

**FIG 1:**
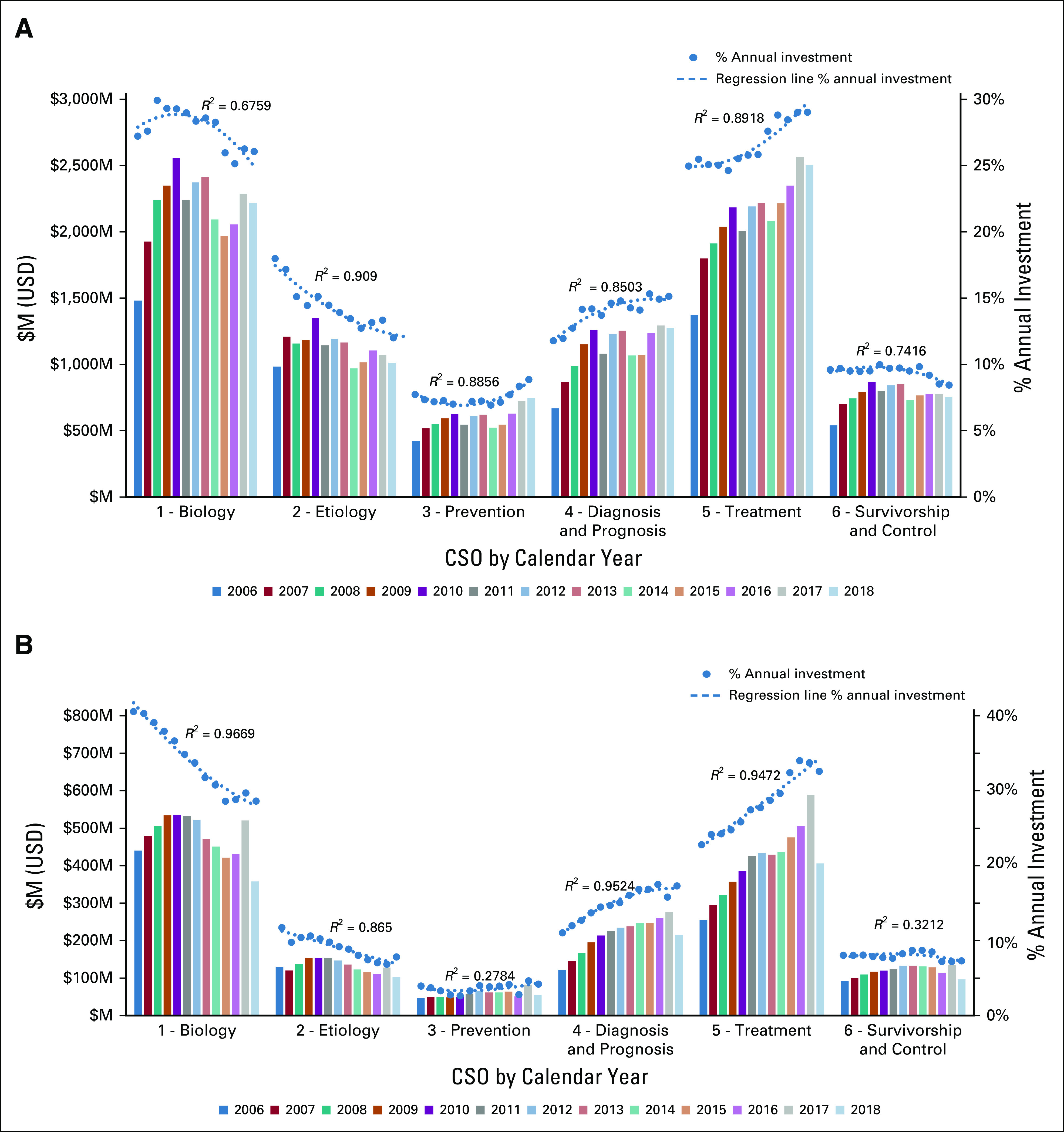
(A) Investment by type of research between 2006 and 2018 (including absolute investment by USD—clustered columns, y axis and as a percentage of the annual portfolio USD value, secondary y axis, and scatter plots with regression lines). (B) Equivalent investment figures by non-NIH partner organizations. CSO, Common Scientific Outline; M, million; NIH, US National Institutes of Health; USD, US dollars.

Analysis by the main focus of research using CSO categories^3^ showed that treatment (CSO 5, 29%) was the largest investment category in 2018, followed by Cancer Biology (CSO 1, 26%); Early Detection, Diagnosis, and Prognosis (CSO 4, 15%); Etiology (CSO 2, 12%); Control, Survivorship, and Outcomes (CSO 6, 9%); and Prevention (CSO 3, 9%) (Fig 1A). As the US NIH was the largest contributor to the data set, trends for all other organizations were analyzed separately (Fig 1B), showing similar patterns to NIH data: Treatment (CSO 5, 33%) was the largest category, followed by Cancer Biology (CSO 1, 29%); Early Detection, Diagnosis, and Prognosis (CSO 4, 17%); Etiology (CSO 2, 8%); Control, Survivorship, and Outcomes (CSO 6, 8%); and Prevention (CSO 3, 5%).

**FIG 2:**
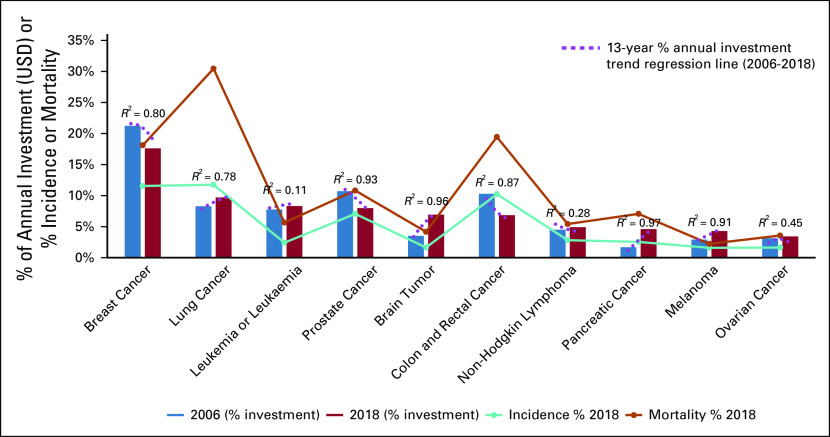
Cancer site: change in % investment^a^ between 2006 and 2018 versus incidence and mortality % (2018)—major sites by investment. ^a^For clarity, individual years’ data points are omitted from the regression line for trends in investment in specific cancer sites (2006-2018) USD, US dollars.

Each project included in the analysis was coded to one or more cancer site(s).^3^ A breakdown of the portfolio by site revealed changes in research funding emphasis from 2006 to 2018. Not site-specific research (ie, research that was not linked to any one specific cancer site or that was equally relevant to all cancers, eg, pain control) accounted for an increased percentage of the portfolio (from 32.3% of overall investment in 2006 to 33.8% in 2018). Analysis of the cancer-site specific research investment (66.2% of the portfolio in 2018) revealed changing patterns in investment. Absolute levels of investment had increased over the period 2006-2018 for all 10 of the cancer sites receiving the highest level of investment in 2018. The results for these cancer sites showed a significant decline in the proportion of funding to breast cancer from 2006 (but an increase in absolute investment from $787M USD in 2006 to $993M USD in 2018) (Fig 2). Funding for colorectal cancer showed only a small increase in absolute terms ($381M USD in 2006 to $385M USD in 2018) and a decline in terms of proportional investment (10.3%-6.8%), dropping from the third highest to sixth highest investment. Similarly, funding for prostate cancer showed a small increase in absolute terms ($397M USD in 2006 to $450M USD in 2018), but a decline in terms of proportional investment (10.7%-8%). By contrast, investment for lung cancer, leukemia, brain cancers, non-Hodgkin lymphoma, pancreatic cancer, melanoma, and ovarian cancer increased both in absolute terms and as a percentage of the portfolio.

**FIG 3:**
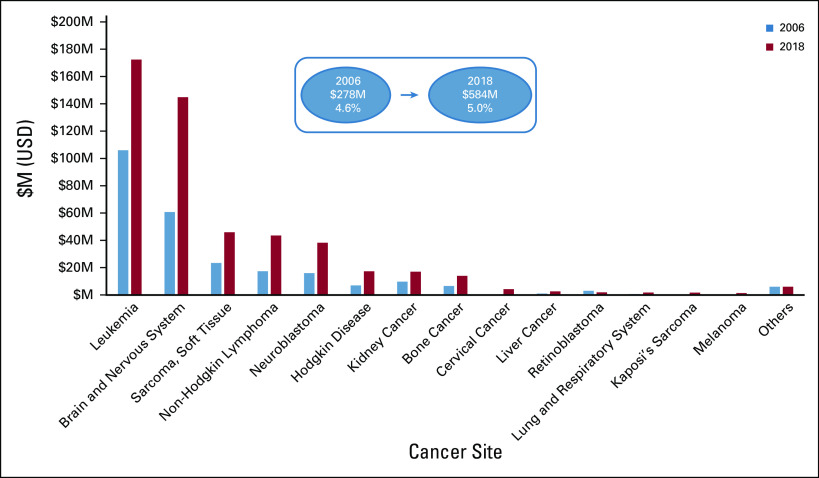
Childhood cancer research—CSO pattern of investment in 2018 for cancer sites of projects wholly and partially related to childhood cancer. CSO, Common Scientific Outline; M, million; USD, US dollars.

Over the period 2006-2018, CYA cancer research accounted for 5% of the annual funding portfolio on average (range, 4.6%-5.8%) and absolute investment had increased from $278M to $584M USD for projects wholly and partially related to CYA cancers. Analysis of the types of cancer researched in CYA cancer projects (Fig 3) demonstrated that leukemia, brain, and sarcoma (soft tissue) were the major research focus areas for projects in the portfolio in 2018.

### Focus on Detailed Trends in Pancreatic Cancer, Prevention Research (CSO 3), and Survivorship Research (CSO 6)

**FIG 4:**
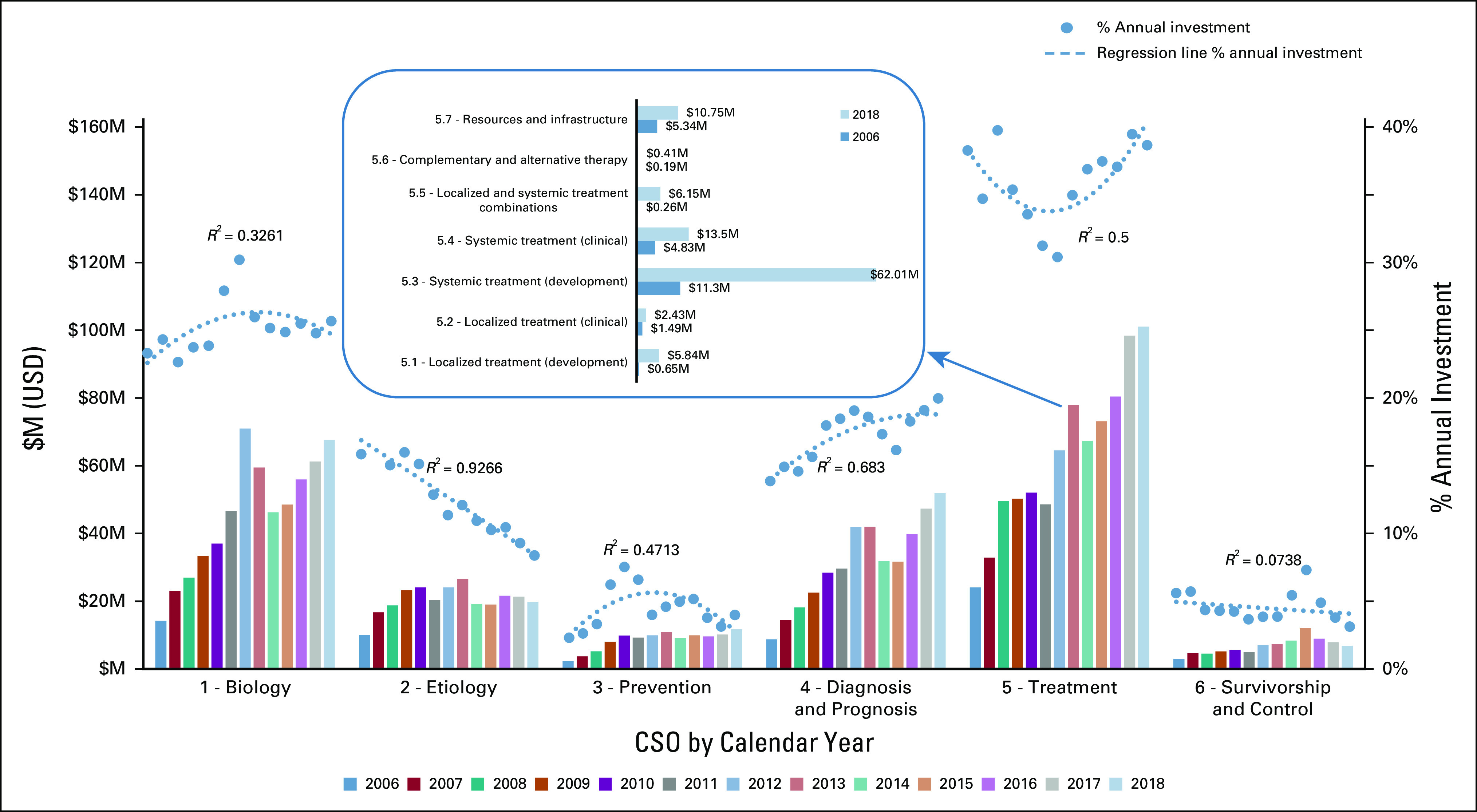
Pancreatic cancer research investment. CSO, Common Scientific Outline; M, million; USD, US dollars.

In this paper, it was not possible to present detailed trends for all cancer types and research types. Detailed data have been made available for analysis using the public data set accompanying this paper, and therefore, in this paper, we chose to focus on the cancer type showing the highest increase in percent investment in this time period (pancreatic cancer) and highlight trends in prevention and cancer survivorship research (the research areas receiving the lowest level of investment overall). Absolute investment in pancreatic cancer research increased threefold between 2006 ($62M USD) and 2018 ($259m USD), as did proportional investment (1.7%-4.6%), with a significantly increased focus on research into diagnosis (CSO 4) and a corresponding reduction in proportional investment in etiology (CSO2) (Fig 4). The increased investment in treatment (CSO 5) was primarily driven by a notable increase in absolute or proportional investment in preclinical discovery and development of systemic therapies (CSO 5.3) (Fig 4, inset).

**FIG 5:**
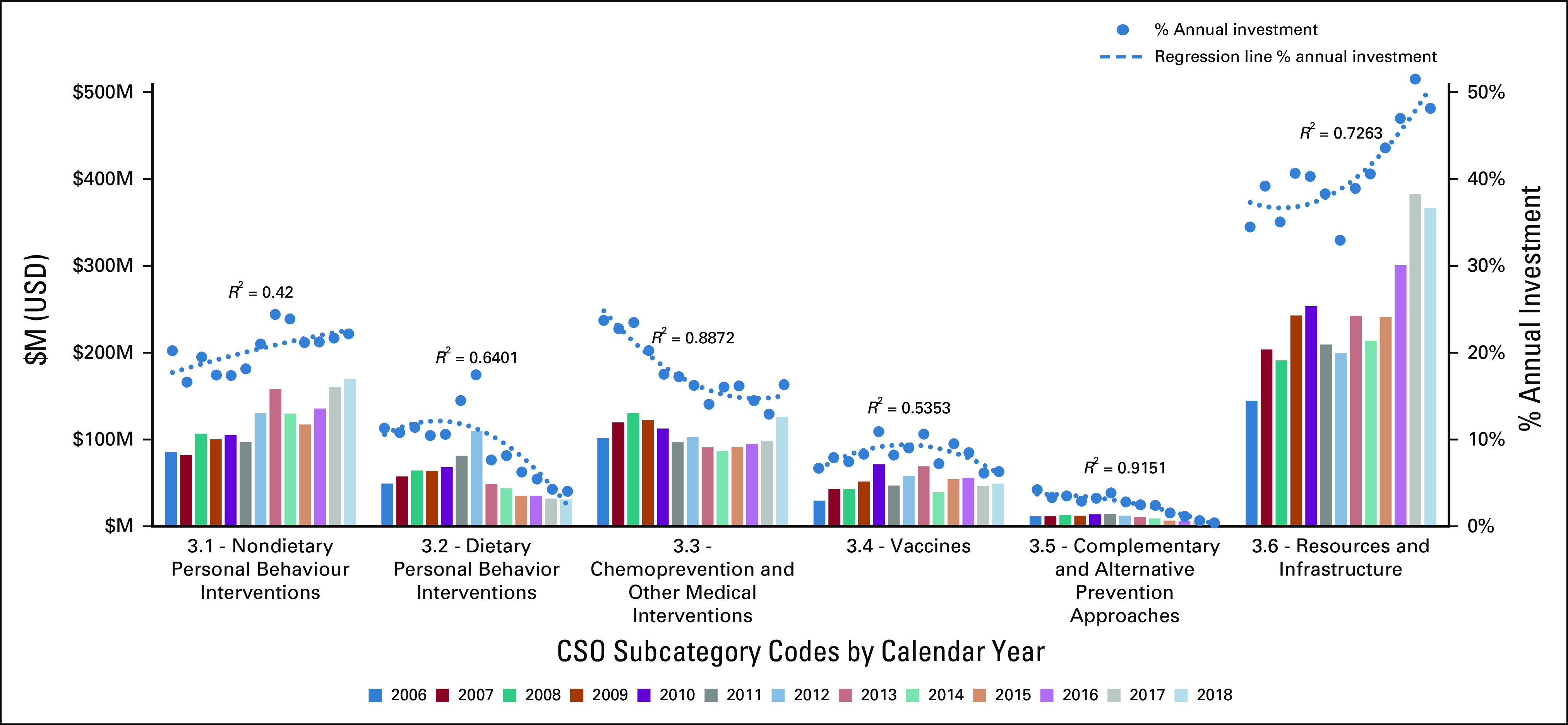
Prevention research. CSO, Common Scientific Outline; M, million; USD, US dollars.

The overall proportion of research investment in prevention (CSO 3) had not changed markedly between 2006 and 2018 (average of 7.5%, range, 7.1%-8.8%). The small upward trend in absolute investment was not statistically significant (*R*^2^ < 0.5, not shown), and the trend in annual percent investment was not conclusive (regression plot, Fig 1A). Increased investment in resources and infrastructure to support prevention was observed (CSO 3.6) in this timeframe, as was research investment in nondietary behavioral interventions (CSO 3.1) (Fig 5).

**FIG 6:**
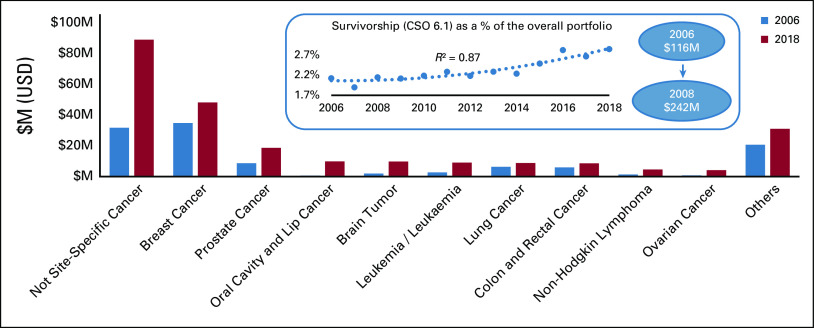
Survivorship research—changes in investment by cancer site between 2006 and 2018. Inset: Survivorship (CSO 6.1) as a percentage of the overall portfolio. CSO, Common Scientific Outline; M, million; USD, US dollars.

As the number of cancer survivors globally is increasing, trends in investment levels were assessed. Absolute levels of investment in survivorship research (CSO 6.1) had increased from $116M USD in 2006 to $242M USD in 2018, and proportional investment rose significantly from 2.1% of the portfolio in 2006 to 2.8% in 2018 (*R*^2^ = 0.87). Most research in this area was not specific to any one cancer site (Fig 6).

## DISCUSSION

In conclusion, ICRP partners' project-level funding data coded to CSO and cancer site provide a unique resource for analyzing current and historic activity in international cancer research investment. Whether data were viewed by absolute investment levels or by percentage of annual investment, the results demonstrated that between 2006 and 2018 funded research had become more translational and clinical in focus (Fig 1A), as shown by statistically significant increases (*R*^2^ > 0.5) in annual proportional investment in CSO 4 (Early Detection, Diagnosis, and Prognosis) and CSO 5 (Treatment). There was a statistically significant reduction in percent investment in CSO 2 (Etiology). Reductions were also observed for percent investment in CSO 1 (Biology) and CSO 6 (Control, Survivorship, and Outcomes). Investment levels in CSO 3 (Prevention) varied between 7% and 9% of the portfolio annually. These trends across CSO categories were similar in the non-NIH partner organizations’ investments (Fig 1B), but with a more marked increase in proportional investment in CSO 4 (Early Detection, Diagnosis, and Prognosis) over this period than in the overall portfolio. The data summarized in Figures 1A and 1B (CSO) provided evidence to support a strategic shift toward more translational research over the period 2006-2018, as a higher proportion of research investment was focused toward the more translational CSO codes.^10^ However, the overall funding distribution across the portfolio in 2018 was similar to that in 2006, in terms of proportional funding awarded to individual CSO codes, and was consistent with proportional funding patterns identified in a previous analysis of funding data from the ICRP database undertaken from 2005 to 2008.^11^ Although investment levels do not necessarily reflect research priorities, the data showed (Fig 2) that cancers having the greatest proportional burden of disease (as assessed by international cancer incidence and mortality statistics) received the highest proportional level of international research investment. There were some notable increases in funding for some cancers such as for pancreatic cancer and brain tumors. Absolute investment in pancreatic cancer research increased threefold between 2006 ($62M USD) and 2018 ($259M USD), as did proportional investment (1.7% in 2006 to 4.6% in 2018), with a significantly increased focus on research into early diagnosis (CSO 4) and a corresponding reduction in proportional investment in etiology (CSO2) (Fig 4). The increased investment in treatment (CSO 5) was primarily driven by a notable increase in absolute or proportional investment in preclinical discovery and development of systemic therapies (CSO 5.3) (Fig 4, inset). A previous analysis of ICRP data^12^ had highlighted the importance of both the government and the nonprofit sector in promoting research activity for this cancer, and the present analysis has shown that significant additional investment has been made. Similarly, absolute investment in research into brain tumors more than doubled, from $130M USD in 2006 to $391M USD in 2018, as did proportional investment (3.5% in 2006 to 6.9% in 2018), responding to challenge of addressing the ongoing and unchanging high levels of mortality for these cancers and the need for additional research investment.

Investment in projects wholly related to childhood cancer had increased from $144M USD in 2006 to $328M USD in 2018; however, there was no evidence for a significant increase in annual proportional investment as a percent of the overall portfolio. Increased investments in 2015-2018 reversed the downward trend from 2012-2014 (not shown) and the downward trend reported by Loucaides et al^13^ for 2011-2016. Most of the increase in 2015-2018 was due to increased funding in the government sector (representing 90% of all investment), but investment in the nonprofit sector also more than doubled (from $25m USD in 2006 to $58m USD in 2018). Although funding for CYA is proportional to the incidence of disease overall, it will be important to ascertain if it is proportional to other measures of disease burden, such as quality-adjusted life year or disability-adjusted life year. A detailed analysis of trends in international childhood cancer research funding will be the focus of a future publication.

Despite the increasing importance of primary prevention in addressing the global cancer burden, the overall proportion of research investment in this area had not changed markedly between 2006 and 2018. As the small upward trend in absolute investment was not statistically significant (*R*^2^ < 0.5, not shown) and the trend in annual proportional investment was not conclusive (Fig 1A), it will be important to track whether the increased investment in resources and infrastructure in prevention research (CSO 3.6) leads to increased primary prevention research in the future.

Survivorship research will also be of increasing importance, as treatment improvements lead to an increasing number of cancer survivors. Survivorship (CSO 6.1) encompasses a wide range of research on living with and beyond cancer, from side effects to pain control, and supportive care for survivors and caregivers. This analysis showed that between 2006 and 2018, there was a small uplift in funding (*R*^2^ = 0.87) in this area. A future analysis will focus on text-mining research abstracts in this area to assess if funded research correlates with patient and survivor research priorities.

ICRP includes all cancer-related data from the world’s largest cancer research funder (US National Institutes of Health), consortia covering the major funders in the United Kingdom and Canada, and other major funding organizations across the world. Estimates of ICRP's coverage of the world governmental and nonprofit investment in cancer research over 2006-2018 have ranged from 60% to 65%. Nonpartner funding totals were estimated using two sources: published annual financial reports of major nonpartner organizations named in the International Agency for Research into Cancer's (IARC) list of world cancer research funders^14^ and publication volume for partner and nonpartner organizations using Web of Science as a proxy for research investment^15^ (however, analysis suggested that although high publication volume indicated higher research investment levels, the correlation was not sufficiently robust to infer nonpartner funding amounts directly).

Understanding the research funding landscape is key to making informed funding decisions. Previous analyses of the research funding landscape have led to strategic decisions by partners to promote research activity in key gap areas, either in partnership or unilaterally. For example, an analysis of research into environmental influences in breast cancer^16^ raised concern over the general decline in research funding in this area. Using these data and other evidence, a partner organization (California Breast Cancer Research Program) launched a call for proposals for additional research and capacity building in this area.^17^

ICRP partner organizations plan to analyze areas with low investment (or where investment is proportionally low in comparison with tumor burden) in more depth to understand barriers to research progress. ICRP partners are working together to analyze three areas in more depth, namely, barriers to research progress in childhood cancer (focusing on cancers with a low survival rate), prevention (understanding barriers to implementation), and survivorship (primarily focusing on whether research activity correlates with patient priorities). As research funders may wish to conduct their own in-depth analyses of areas of research funding, to accompany this publication, ICRP has provided an online cancer research investment data pack ^18^ to view trends in CSO and cancer site research funding profiles and geographical investment year-on-year between 2006 and 2018.

ICRP's motivation in providing cancer research funding landscape analyses is to give researchers the tools to avoid duplication of research, identify potential collaborators, and give organizations the capacity to make informed, strategic research funding decisions based on an understanding of the overall context of international cancer research funding, especially in the current situation where the budgets of many organizations are being affected by COVID-19. Our recent survey of 86 international cancer research funders indicated that 66% of respondents from charitable or private foundations expected that their funding available for cancer research would decrease significantly in 2020 and 2021.^19^ Although charities or private funders make up only around 7%-10% of the ICRP portfolio, any reduction in investment from this sector will be significant globally. This analysis provides a baseline from which the potential impact of reduced research budgets because of COVID-19 can be measured. We anticipate that a full analysis of the impact of COVID-19 on research investment in 2020 and 2021 will be possible in 2023.

The ICRP partners welcome additional data from other funders to help complete the picture of global cancer research funding and to help ensure that cancer research investments are evidence-based and focused strategically at an international level.
